# The cognitive effects of listening to background music on older adults: processing speed improves with upbeat music, while memory seems to benefit from both upbeat and downbeat music

**DOI:** 10.3389/fnagi.2014.00284

**Published:** 2014-10-15

**Authors:** Sara Bottiroli, Alessia Rosi, Riccardo Russo, Tomaso Vecchi, Elena Cavallini

**Affiliations:** ^1^Brain Connectivity Center, National Neurological Institute C. MondinoPavia, Italy; ^2^Brain and Behavioral Sciences Department, University of PaviaPavia, Italy; ^3^Department of Psychology, University of EssexEssex, UK

**Keywords:** background music, arousal, mood, processing speed, episodic memory, semantic memory, aging

## Abstract

Background music refers to any music played while the listener is performing another activity. Most studies on this effect have been conducted on young adults, while little attention has been paid to the presence of this effect in older adults. Hence, this study aimed to address this imbalance by assessing the impact of different types of background music on cognitive tasks tapping declarative memory and processing speed in older adults. Overall, background music tended to improve performance over no music and white noise, but not always in the same manner. The theoretical and practical implications of the empirical findings are discussed.

## Introduction

Background music refers to any music that is played while the listener’s primary attention is focused on another task or activity (Radocy and Boyle, 1988). This background music effect differs from the so-called Mozart effect (Rauscher et al., 1993), which refers to the changes in cognitive abilities following listening to music. Despite the above difference, there is consensus on these effects to operate on common mechanisms (Schellenberg and Weiss, 2013). The present study primarily focused on the background music effect.

Some studies on the effect of background music on performance in cognitive tasks have shown improvements in episodic memory (Ferreri et al., 2013), IQ scores (Cockerton et al., 1997), verbal and visual processing speed (Angel et al., 2010), arithmetic skill (Hallam and Price, 1998), reading (Oliver, 1997), and second languages learning (Kang and Williamson, 2013). However, there is also evidence of reduced performance when background music is present (see Kämpfe et al., 2010 for a review).

According to the “arousal and mood hypothesis” (Thompson et al., 2001), the positive effect of music on human behavior is considered to be a consequence of the impact of music on mood and arousal. In particular, listening to music affects arousal (degree of physiological activation), mood (long lasting emotions), and listener’s enjoyment, which in turn influence cognitive performance (Hallam et al., 2002). The impact of music on arousal and on mood of listeners seems to be determined by the tempo (fast vs. slow) and the mode (major vs. minus) of the music itself, respectively (Gabrielsson and Lindström, 2010). In particular, as reported in the context of the Mozart effect, fast tempo and major mode music tend to induce a positive/happy mood and higher arousal levels, whereas slow tempo and minor mode music induce a more negative/sad mood and lower arousal levels (e.g., Husain et al., 2002; Hunter and Schellenberg, 2010). Moreover, the effects of these different levels of mood and arousal seem to vary depending on the cognitive abilities considered. In particular, several studies investigating the Mozart effect reported benefits primarily using tasks tapping processing speed and visuo-spatial abilities but only when the music had a fast tempo and a major mode (e.g., Thompson et al., 2001; Husain et al., 2002; Schellenberg et al., 2007).

Conversely, disturbing and interfering effects of background music have been reported for multimedia learning (Moreno and Mayer, 2000), surgeons learning of new procedures (Miskovic et al., 2008), mathematics (Bloor, 2009), and reading (Madsen, 1987). These negative findings could be explained by the “cognitive-capacity hypothesis” (Kahneman, 1973), positing that a limited pool of resources is available for cognitive processing at any given moment (Baddeley, 2003), thus background music can disrupt cognitive tasks when there is a potential for interference (e.g., Polzella and Schoeling, 2004) due to an overtax of resources (Norman and Bobrow, 1975). In particular, detrimental effects related to background music seem to be modulated by task complexity: the more complex and demanding the task, the stronger is the detrimental effect of music (Furnham and Bradley, 1997; Furnham and Allass, 1999). In summary, with respect to the background music effect there are conflicting results as well as conflicting theoretical approaches that may, in principle, provide a unified account of the effect (or lack of it) on the basis of task complexity. When task complexity surpasses some critical threshold, then performance is impaired. Conversely, below a certain level of task complexity the arousal and mood hypothesis may account for some beneficial effects of background music on task performance. While this theoretical stance may be appealing, it is not clear why background music, below certain levels of task complexity, it is not simply neutral, but it is indeed beneficial.

An interesting way to test the potential merit of the above hypotheses consists of assessing the background music effect on older adults. Given that normal aging is particularly associated with deficits in inhibiting irrelevant information and with deficits in tasks performed under divided attention (e.g., Parks, 2007), background music should negatively affect performance in cognitive tasks in older adults. Hence, if background music does provide a beneficial effect to performance in cognitive tasks in older adults, then the validity of the “cognitive capacity hypothesis” would be weakened. Therefore, the present study intended to assess the impact of background music on the performance of older adults in cognitive tasks.

To the best of our knowledge, only three studies had been conducted on normal aging (Thompson et al., 2005; Mammarella et al., 2007; Ferreri et al., 2014). These compared the effects of listening to music excerpt with high tempo and major mode vs. no-music on word fluency (Thompson et al., 2005; Mammarella et al., 2007), on working memory (Mammarella et al., 2007), and on recognition memory (Ferreri et al., 2014). Interestingly, they all reported a specific positive effect of the background music that was able to enhance performance on the cognitive abilities examined. However, these studies in failing to include a negative emotional-valence background music (low tempo in minor mode), did not provide a thorough assessment of the impact of background music on cognitive tasks. Hence, in the present study we included two different types of background music. We selected Mozart’s Eine Kleine Nachtmusik (positive background music with fast tempo and major mode) and Mahler’s 5th Symphony Adagietto (negative background music with slow tempo and minor mode), on the basis that these two pieces of music have been shown to induce happy and sad moods, and high and low arousal levels, respectively (Niedenthal and Setterlund, 1994; Storbeck and Clore, 2005; Riener et al., 2011). Furthermore, we also used two control conditions: a no-music and a white noise control conditions to assess whether music improves (impairs) performance over baseline conditions. In particular, the white noise refers to a special type of environmental stimulation consisting in the exposure to a continuous auditory signal. Previous evidences on white noise have produced mixed findings, reporting some instances of disturbing effects due to a competition for cognitive resources (e.g., Hygge et al., 2003; Boman et al., 2005) and others demonstrating that it was able to promote learning in those subjects with attentional deficits thanks to an increase of arousal levels (e.g., Söderlund et al., 2007, 2010).

In order to evaluate the effects of background music on different cognitive abilities, we used tests tapping processing speed and declarative memory. Our decision was driven by three main reasons. Firstly, processing speed is one of those abilities sensitive to the tempo and the mode of the music in those studies involving students (e.g., Schellenberg et al., 2007; Angel et al., 2010), thus it could represent a clear probe of the possible different effects of positive and negative background music in older adults. Second, the effect of background music on memory is rather controversial in the literature on young adults, with evidences of both beneficial effects (e.g., Ferreri et al., 2013) and detrimental effects (e.g., Moreno and Mayer, 2000; Miskovic et al., 2008). Hence, we intended to assess the impact of different types of background music on tests tapping what are nominally called episodic memory (free recall) and semantic memory (phonemic fluency). Third, both processing speed and memory are cognitive abilities mostly affected by aging (see Salthouse, 2004), thus it is of interest to assess whether background music may have a negative or positive effects on these tasks among older adults.

Because of the above theoretical considerations, some speculations could be put forward. On one hand, if older adults are sensitive to the “arousal and mood” effect of music (Thompson et al., 2001), their performance should be enhanced by background music in comparison to the two control conditions (no-music and white noise) with different effects between the positive and the negative condition. With respect to processing speed, performance should improve while listening to the fast tempo and major background music compared to a slow tempo and minor mode background music (e.g., Schellenberg et al., 2007). With respect to memory, prior research suggested that fast tempo and major mode background music should improve performance in the elderly (Mammarella et al., 2007; Ferreri et al., 2014). To the best of our knowledge the effect of a slow tempo and minor mode background music on memory among older adults has not been investigated.

On the other hand, according to the “cognitive-capacity hypothesis” (Kahneman, 1973), we should expect that in the tasks used performance among older adults when exposed to no-music will be significantly greater than in the other experimental conditions.

## Material and method

### Participants

Sixty-five non musicians older adults took part in the study. Their mean age was 69.03 (*SD* = 5.79; age range 60–84 years; 51 females and 14 males) and mean of education was 12.29 years (*SD* = 3.88). Exclusion criteria included history of psychiatric or neurological disorders, substance abuse and a score of 23 or higher on the Center for Epidemiological Studies Depression Scale (CES-D; Radloff, 1977; Italian version, Fava, 1983). None of the participants was excluded on the basis of the above criteria. A Vocabulary test (PMA; Thurstone and Thurstone, 1963) was also included in the study to assess crystallized intelligence. They had a mean score of 45.69 on Vocabulary test (*SD* = 3.67) out of a maximum of 50, and a mean score of 15.56 on the CES-D (*SD* = 6.83). All participants completed and accepted an informed consent form prior to the beginning of the experiment.

### Instruments

All participants undertook the Vocabulary subtest and the CES-D as control variable measures and three paper-and-pencil cognitive tasks to assess, respectively, declarative memory (episodic memory and semantic memory) and processing speed.

#### Vocabulary

In the Vocabulary subtest participants were asked to identify the correct synonym of 50 target words within 8 min. Total scores could range from 0 to 50.

#### Depression

The CES-D consisted of 20 multiple-choices questions, asking participants to rate the frequency with which they have experienced depressive symptoms during the past week. Responses for each questions range from 0 (*rarely or none of the time*) to 3 (*most or all of the time*). Possible scores could range from 0 to 60.

#### Declarative memory

*Episodic memory*. Participants were presented with a list of 15 concrete words in order to assess the episodic memory. Lists of word were presented visually on the screen. Participants were instructed to commit to memory the list in a study period of 2 min and, immediately after presentation, were given 2 min to write down, in any order, as many words as could they remembered. We developed four parallel versions of the words-lists in order to assess the same task in the four different conditions. The words were taken from Paivio et al. (1968) words norms and the four parallel versions did not significantly differ in term of imagery, concreteness and frequency of use. The episodic memory score used was obtained by subtracting the intrusion words from the number of correctly recalled words.

*Semantic memory*. To assess semantic memory, a phonemic fluency task was used. Participants were asked to write down on an answer sheet, as many words as possible beginning with three different letters of the alphabet. They were instructed that proper names, place and words with the same suffix were not credited. We developed four parallel versions of the phonemic fluency task, by changing the starting letters, in order to assess the same task in the four different conditions. Participants were given 90 s for each letter, and semantic memory score used was obtained by subtracting the words erroneously produced (e.g., proper nouns and repetition) from the number of correct words.

#### Processing speed

To assess processing speed in the visual modality, the Symbol Digit Modalities Test (SDMT; Smith, 1982; Italian version, Nocentini et al., 2006) was used. At the top of an A4 sheet of paper, nine abstract geometric shapes were associated with the digits 1–9. Participants had been instructed to write the digit, as quickly as possible, corresponding to the appropriate symbol into rows of empty boxes with symbols above them. In order to assess the same task in the four different conditions, we developed four parallel versions of the SDMT, by changing the associations between digits and symbols. Participants were given 90 s to fill as many empty boxes as possible. Scores for this task were obtained by subtracting the errors from the number of correctly filled boxes.

#### Mood questionnaire

To assess how participants rated positive and negative emotions induced by Mozart’s, Mahler’s and the white noise backgrounds, participants completed for each ones a brief mood questionnaire. The questionnaire included three items assessing: (1) how much participants evaluated the music as happy; (2) how much they evaluated the music as sad; and (3) how much the amount of emotion experienced to listen each music, using a 10-point rating-scale anchored at 1 (very *little*) and 10 (very *much*).

### Procedure

Participants performed the three cognitive tests in all of the four different background conditions: (1) no-music; (2) white noise; (3) Mozart’s *Eine Kleine Nachtmmusik* aimed to induce a positive emotion/mood and high arousal levels; and (4) Mahler’s *Adagietto Symphony 5* aimed to induce a negative emotion/mood and lower arousal levels. Participants were tested in groups of about 10 in two 2-h sessions a week apart. The type of backgrounds used (no-music, white noise, Mozart, Mahler) were counterbalanced across participants, so that half of participants were randomly allocated to the condition where first listened to classical music (either Mozart or Mahler) and then to either white noise or no-music condition, and for the other half of participants the order was reversed. In addition, within each background conditions, the order of the cognitive tests was counterbalanced across participants and subjects were randomly allocated to different tasks order.

In the first session all participants completed, in order, (a) the informed consent form; (b) demographic questionnaire; (c) Vocabulary test; (d) CES-D. Subsequently, both in the first and in the second sessions, they performed, for each background conditions, the parallel versions of the three cognitive tests. In this way, participants performed the same tasks in the four different background conditions. For example, half of participants in the first session performed the cognitive tests listening first to Mozart then the no-music condition (or first listening to Mahler and to white noise secondly), and in the second session performed the cognitive tests listening first to Mahler and then to white noise (or first Mozart followed by the no-music condition). Instead, in the first session, the other half of participants performed the cognitive tests listening first to white noise and secondly to Mozart (or first listening to white noise and to Mahler secondly) and, in the second session, performed the cognitive tests first with no-music in the background and then listening to Mahler (or first the no-music condition followed by Mozart).

Background music was played during the entire task, i.e., before and during tasks’ execution. For this reason, the two classical music and the white noise audio tracks started 1 min before each task, continued during the task, and ended as soon as each task ended. In order to make the experimental conditions comparable, in the silence condition too, participants were asked to be silent for 1 min before starting and during the execution of each task. Before the start of each background condition the experimenter verbally provided the instructions and the entire procedure to the participants. Finally, at the end of the second session, participants completed the mood questionnaire for Mozart, Mahler and white noise backgrounds.

### Statistical analysis

We conducted a series one-way Analyses of Variance (ANOVAs) with background conditions (4 levels: Mozart, Mahler, white noise, and no-music) as a within-subjects factor. A significant level of 0.05 was adopted. Paired *t*-test were used to follow-up significant *F* ratios. Since there were at most six pairwise comparisons, the significance level adopted for these follow-up tests was 0.008, unless otherwise stated. Mean values and standard deviations for the cognitive tasks are reported in Table 1.

**Table 1:** **Means value and standard deviations for cognitive task as a function of background conditions**.

	Background condition			
	Mozart	Mahler	White noise	No-music
**Cognitive tasks**				
Episodic memory	9.82 (2.41)^ab*^	9.92 (2.38)^*cd*^	9.11 (2.32)^*bd*^	8.71 (2.45)^*ac*^
Semantic memory	41.61 (11.55)^*ab*^	39.80 (9.78)^*c*^	36.39 (11.42)^*bc*^	38.34 (9.22)^*a*^
Processing speed	38.89 (10.31)^*abc*+^	35.51 (9.45)^*b*^	34.65 (11.17)^*a*^	36.76 (11.63)^*c*+^

Note: Scores in parenthesis refer to Standard Deviation. For each row, same supra-scripts indicate significant differences at an alpha level of 0.008. * p = 0.014; ^+^ p = 0.068.

## Results

### Cognitive performance

#### Declarative memory

Results on *episodic memory* showed a significant main effect of background conditions, *F*_(3, 192)_ = 7.68, *MSE* = 2.85, *η*^2^ = 0.11. Pairwise comparisons showed a significant advantage for the Mozart condition over no-music, *t*_(64)_ = 3.64, and a marginally significant increase over the white noise, *t*_(64)_ = 2.53, *p* = 0.014. A significant advantage was also found for the Mahler condition over no-music,* t*_(64)_ = 4.01, and the white noise condition, *t*_(64)_ = 3.24. The white noise did not differ from no-music,* t*_(64)_ = 1.14, as well as the Mozart condition from the Mahler condition, *t*_(64)_ = 0.39. Overall, episodic memory performance increased when classical music was played in the background compared to white noise and no music conditions. No significant difference emerged between the two classical music conditions nor between the two non-music conditions.

From the analysis of *semantic memory*, emerged a significant main effect of background condition, *F*_(3, 192)_ = 9.70, *MSE* = 32.95, *η*^2^ = 0.13. Follow-up analyses revealed a significant advantage of the Mozart condition over no-music, *t*_(64)_ = 3.02, and white noise, *t*_(64)_ = 5.21. Performance in the Mahler condition was significantly higher than in the white noise condition,* t*_(64)_ = 3.36. The Mahler condition neither differ significantly from the no-music condition, *t*_(64)_ = 1.93, nor from the Mozart condition, *t*_(64)_= 2.07. Finally, there was not significant difference between the two control conditions,* t*_(64)_ = 1.58. Overall, listening to classical music increased semantic memory performance compared to white noise and no-music. The overall pattern of the impact of the independent variable on semantic memory is comparable to the one obtained in the free recall task.

To further assess whether the effects of background music on episodic and semantic memory were comparable, we carried out a repeated-measure factorial ANOVA 4 (background condition: Mozart, Mahler, white noise, and no-music) as within-subject variable by 2 (task: episodic memory vs. semantic memory) as between-subject variable. Given that the two tasks utilized different scoring options, the dependent variable was the z-score calculated for each subjects for each background music conditions for the episodic and semantic memory tasks (so each participant had a mean of zero). Means and standard deviations for the two declarative memory tasks performance are provided in Table 2. The analysis showed a significant main effect of background condition, *F*_(3, 192)_ = 12.62, *MSE* = 0.65, *η*^2^ = 0.16. It can be noticed from Table 2 that this reflects higher memory performance in the music conditions compared to the no-music conditions. The significant interaction, *F*_(3, 192)_ = 2.96, *MSE* = 0.52, *η*^2^ = 0.04, is not particularly interesting since it is simply a consequence of larger scores for Mahler (0.28) than Mozart (0.23) in episodic memory, while the reverse occurred in the semantic memory task (0.08 vs. 0.28, respectively). Similarly higher scores were associated with the white noise conditions (−0.15) than the no-music condition (−0.36) in the episodic memory task, while the reverse occurred in the semantic memory task (−0.29 vs. −0.08, respectively).

**Table 2:** **Means value and standard deviations for the *z*-score of the two declarative memory tasks (episodic and semantic memory) as a function of background conditions**.

	Background condition			
	Mozart	Mahler	White noise	No-music
**Declarative memory tasks**				
Episodic memory	0.23 (1.27)	0.28 (1.26)	−0.15 (1.22)	−0.36 (1.30)
Semantic memory	0.28 (1.24)	0.08 (1.05)	−0.29 (1.23)	−0.08 (0.99)

*Note: Scores in parenthesis refer to Standard Deviation*.

#### Processing speed

Regarding the SDMT, the main effect of background conditions was significant, *F*_(3, 192)_ = 5.70,* MSE* = 37.06, *η*^2^ = 0.08. Follow-up analyses showed a significant advantage of the Mozart condition over the white noise, *t*_(64)_ = 4.34, the Mahler condition, *t*_(64)_ = 3.75, and an advantage over the no-music condition that did not reach significance, *t*_(64)_ = 1.86. The Mahler condition neither differ significantly from the white noise, *t*_(64)_ = 0.90, nor from the no-music condition, *t*_(64)_ = 1.20. Finally, there was not significant difference between two control conditions, *t*_(64)_ = 1.51. Overall, it appears that the Mozart condition was associated with the highest performance and that this condition seemed to differ from the others which were comparable.

### Mood questionnaire

Finally, to ensure that the two classical music induced positive and negative emotions, we analyzed scores obtained by the brief mood questionnaire for Mozart, Mahler and white noise backgrounds. To this end, we conducted a series one-way ANOVAs with background conditions (3 levels: Mozart, Mahler, and white noise) as a within-subjects factor. Paired *t*-test were used to follow-up significant *F* ratios. For technical reasons a smaller than the full sample provided the mood ratings.

Results on how music was evaluated as *happy* showed a significant main effect of background conditions, *F*_(2, 52)_ = 236.72, *MSE* = 1.65, *η*^2^ = 0.90. Pairwise comparisons showed that Mozart’s excerpt was considered happier than Mahler’s Adagietto (*M*_Mozart_ = 8.63; *M*_Mahler_ = 2.33),* t*_(26)_ = 18.24, and than the white noise condition (*M*_white noise_ = 1.78), *t*_(26)_ = 18.21. Mahler and the white noise condition did not differ significantly, *t*_(26)_ = 1.70.

From the analysis of how music was evaluated as *sad*, a significant main effect of background condition emerged, *F*_(2, 52)_ = 19.78, *MSE* = 5.95, *η*^2^ = 0.43. Follow-up analyses revealed that Mahler was rated as more sad than Mozart (*M*_Mozart_ = 1.41; *M*_Mahler_ = 5.41), *t*_(26)_ = 7.61, but comparable to white noise condition (*M*_white noise_ = 4.44), *t*_(26)_ = 1.27, Finally, the white noise condition was rated more sad than Mozart (*M*_Mozart_ = 1.41), *t*_(26)_ = 4.42.

Regarding the assessment of *emotion*, the main effect of background condition was also significant, *F*_(2, 52)_ = 79.88, *MSE* = 3.69, *η*^2^ = 0.75. Follow-up analyses showed that participants reported that Mozart induced greater emotionality than Mahler (*M*_Mozart_ = 8.70; *M*_Mahler_ = 6.89), *t*_(26)_ = 3.69, and the white noise conditions (*M*_white noise_ = 2.30), *t*_(26)_ = 12.50. Finally, Mahler was significantly different from the white noise condition, *t*_(26)_ = 8.19.

## Discussion

The present study examined the impact of different types of background music on tasks tapping declarative memory and processing speed in a sample of healthy older adults. From the obtained results it appeared that performance in the processing speed task, was enhanced when Mozart’s music was played in the background compared to the other three conditions, Mahler included. Conversely, background music affected differently memory performance. Unlike the processing speed task, in the case of episodic and semantic memory tasks both “positive” and “negative” background music conditions induced a significant performance advantage over the silence and white noise conditions, which did not significantly differ in their effect.

The results obtained in the processing speed task could, in principle, be accommodated by the “arousal and mood hypothesis” (Thompson et al., 2001) since performance increased systematically in the condition that induced increased positive mood and arousal. However, the above result in conjunction with those obtained in the memory tasks do not appear to support the view that increased positive mood and arousal necessarily lead to improved performance. Indeed, free recall and phonemic fluency also benefitted from a background music inducing a more negative mood. Hence, we cannot consider valid those approaches retaining that background music leads to improved performance only if it increases positive mood and arousal levels.

A *post hoc* interpretation may consist in retaining that background music improves performance when the mood and arousal induced by the music are optimal to support the processes involved in the cognitive task being performed. For example, processing speed tasks, due to their nature, may benefit more from a more alert mood and state. These tasks comprise a motor tracking valence that could have benefitted from a synchronization between movement and auditory rhythms (McElrea and Standing, 1992; Repp and Penel, 2004). The fast and major music could have in turn produced the best condition to support older adults’ performance, as reported by other research (e.g., Konz and McDougal, 1968; McElrea and Standing, 1992; Repp, 2006).

Conversely, memory tasks may be positively affected by any type of background music inducing emotive states. The fact that music is able to evoke emotions is well known (e.g., Panksepp, 1995). For instance, Sacks (2006) refers to the evocative power of music in evoking transcendent emotions. Given that emotions enhance memory processes and music evokes strong emotions (Jäncke, 2008), our findings highlight the possibility that any type of background music can facilitate memory performance. An explanation is that music activates the limbic system, which is involved in processing the emotions and in controlling memory (e.g., Blood et al., 1999). Evidence supporting this come from those studies using therapeutically music to enhance memory in Alzheimer’s disease patients by provoking emotional responses (e.g., El Haj et al., 2012).

A further consideration may be made. If emotional experience comprises two dimensions, valence and intensity (e.g., Duffy, 1941), our data seem to suggest that the effects of music on memory are due primarily to the intensity of the emotions induced by music, rather than their specific valence. This purely *post hoc* speculation obviously requires empirical testing before being considered as a valid explanation of the background music effect.

Furthermore, it is relevant to notice that a context dependent learning effect (e.g., Smith, 1985) should not be considered as a valid explanation of the results obtained in the memory tasks for two reasons. Firstly, in the white noise condition, as well in the music conditions, some comparable background sound was presented during the study and test phase of the free recall task. Nevertheless, performance was significantly lower than in the classical background music conditions. Secondly, due to the nature of the semantic memory task, there were no distinctive study and retrieval phases. Despite this feature of the task, classical music in the background led to the highest performance.

Given the cognitive changes associated to normal aging (e.g., Parks, 2007), the lack of a disruptive effect of background music on task performance in our older adults sample seems at variance with the “cognitive-capacity hypothesis” (Kahneman, 1973). According to this view, background music should have negatively affected cognitive performance in older adults. This clearly was not the case in the present study.

It is important to note that our background music conditions did not involve information that could have directly competed with processing target information (e.g., the music was instrumental and it was not aversively loud), hence the lack of interference we observed. For instance, if cognitive tasks involve the same auditory channel (e.g., Moreno and Mayer, 2000) of the background music, the likelihood of interference should increase, in contrast to stimuli processed in separate channels.

In summary, the different patterns of results found for processing speed and memory seem to suggest that the influence of music is not homogeneous. The impact could depend on the kind of music background but also on features of the task and/or abilities involved. Future studies should better clarify this issue evaluating the impact of background music on cognitive abilities using different channels (e.g., visual vs. verbal) and possibly using tasks marked by increasing levels of complexity. Clearly the background music effect is in need of a valid theoretical explanation.

This study had two main strengths. First, it evaluated the impact of background music on the aging population. Second, it used a more thorough methodology than the one used in previous studies in this field (Thompson et al., 2005; Mammarella et al., 2007; Ferreri et al., 2014).

Finally, considering a more pragmatic approach, the results of this study provide a positive message. Listening to music could indeed represent a relatively inexpensive and non-invasive method to enhance those cognitive abilities that are crucial to the daily living in elderly adults.

## Conflict of interest statement

The authors declare that the research was conducted in the absence of any commercial or financial relationship that could be construed as a potential conflict of interest.
